# The Mammalian Circadian Timing System and the Suprachiasmatic Nucleus as Its Pacemaker

**DOI:** 10.3390/biology8010013

**Published:** 2019-03-11

**Authors:** Michael H. Hastings, Elizabeth S. Maywood, Marco Brancaccio

**Affiliations:** 1 MRC Laboratory of Molecular Biology, Division of Neurobiology, CB2 0QH Cambridge, UK; emaywood@mrc-lmb.cam.ac.uk; 2 UK Dementia Research Institute at Imperial College London, Division of Brain Sciences, Department of Medicine, W12 0NN London, UK; m.brancaccio@imperial.ac.uk

**Keywords:** astrocytes, entrainment, photoperiod, suprachiasmatic, period, cryptochrome, sleep, clock, Bmal1

## Abstract

The past twenty years have witnessed the most remarkable breakthroughs in our understanding of the molecular and cellular mechanisms that underpin circadian (approximately one day) time-keeping. Across model organisms in diverse taxa: cyanobacteria (*Synechococcus*), fungi (*Neurospora*), higher plants (*Arabidopsis*), insects (*Drosophila*) and mammals (mouse and humans), a common mechanistic motif of delayed negative feedback has emerged as the *Deus ex machina* for the cellular definition of ca. 24 h cycles. This review will consider, briefly, comparative circadian clock biology and will then focus on the mammalian circadian system, considering its molecular genetic basis, the properties of the suprachiasmatic nucleus (SCN) as the principal circadian clock in mammals and its role in synchronising a distributed peripheral circadian clock network. Finally, it will consider new directions in analysing the cell-autonomous and circuit-level SCN clockwork and will highlight the surprising discovery of a central role for SCN astrocytes as well as SCN neurons in controlling circadian behaviour.

## 1. Introduction

To adapt to daily and seasonal environmental cycles, living organisms need to vary their metabolism, physiology and behaviour within strict temporal programmes that tune them to the alternating demands and opportunities of night and day, summer and winter. Consequently, evolution has furnished them with intrinsic biological clocks, which confer the critically important adaptive ability to anticipate, and thereby prepare for, these environmental cycles [1]. This preparative anticipation is evidenced by the bewildering array of overt circadian (and seasonal) rhythms, hard-wired into nature. For example, the photosynthetic apparatus of cyanobacteria and higher plants is up-regulated in advance of dawn to maximise light-harvesting efficiency, whilst circadian activation of the human adrenal gland drives increasing secretion of corticosteroids, also in advance of dawn, ensuring optimal physical and mental performance immediately after waking [2,3]. The intrinsic nature of these rhythms and the clocks driving them is revealed experimentally by testing the effects of temporal isolation of individuals within an unchanging environment. Under such constant conditions, circadian rhythms persist, thereby demonstrating their control by an endogenous timer [4]. As with all biological systems, however, the period of the “free-running” rhythms varies slightly between individuals, and is only approximately, not exactly, one day (hence circa-dian). Under natural field conditions, however, synchronisation of the circadian mechanism to the environment sets the period to exactly 24 h, and also sets the phase of the clock, matching internal time to the appropriate solar time. Typically, entrainment is effected by the cycle of light and darkness, although depending on ecological niche, relevant non-photic cues may also contribute. Importantly, however, circadian clocks and the rhythms they drive are temperature compensated [5,6,7]. Without this unique property they would run faster under higher temperatures and so be unable to predict solar time. Finally, many circadian mechanisms are also able to incorporate photoperiodic information and so can function as a seasonal calendar [8,9], cued by changes in daylength and thereby predicting the seasons.

The pervasive nature of circadian timing begs the question: what are the genetic, molecular and cellular bases of the underlying clock mechanisms? In this review, we consider, briefly, the comparative biology of circadian timing mechanisms across different taxa. We then focus on the circadian system of mammals, a body-wide hierarchy of interlocked circadian oscillators present in all major organs. These are sustained and synchronised to each other and to solar time by the principal circadian pacemaker of the suprachiasmatic nucleus (SCN) of the hypothalamus. We explore how the interplay between cell-autonomous and network-level processes confers on the SCN its remarkable precision and resilience as a biological clock. Finally, we consider the implications of the surprising recent discovery that astrocytes are as important in the control of SCN-driven circadian behaviour as are neurons.

## 2. Circadian Feedback Loops: A Recurrent Motif

### 2.1. Comparative Circadian Clocks: Post-Translational Oscillators

Circadian timing mechanisms have a deep evolutionary history, first arising in prokaryotes such as cyanobacteria. Indeed, cyanobacteria are remarkable organisms: their evolution of photosynthesis led to the creation of chloroplasts and higher plants, but even before then they transformed life on Earth with the Great Oxygenation Event (GOE) [10]. In the extant species *Synechococcus elongatus*, photosynthetic capacity and almost all other aspects of metabolism are strongly circadian and it has proven to be an exceptionally useful model to understand one molecular mechanism for prokaryotic circadian time-keeping. This pivots around a self-sustaining cycle of slow and processive auto-phosphorylation and de-phosphorylation by three proteins, KaiA, KaiB and KaiC. The mechanism, which incorporates a delayed negative feedback loop, is so powerful and elegant, that a simple in vitro mix of the three recombinant proteins and ATP will autonomously and continuously mark daily time, as evidenced by circadian oscillations in the abundance of phospho-KaiC [11,12]. This post-translational timer recruits additional kinases and transcription factors on a circadian basis, and these in turn ultimately direct genome-wide circadian oscillations of gene expression that underpin the daytime- and night-time-specific programmes of metabolism that anticipate solar cycles [13]. In addition, *S. elongatus* also exhibits a second post-translational circadian oscillation that involves the super-oxidation of redox-regulatory peroxiredoxin (PRDX) proteins. This oscillation in redox-regulatory capacity is also seen in the archaeon *Halobacterium salinarum*, identifying its ancient evolutionary origin, which may well have been in response to the challenges posed by increased oxygenation at the GOE [14]. Indeed, circadian PRDX cycles are also highly conserved across eukaryotes, reflecting the deep relationship between clocks and cellular metabolism: remarkably, they are sustained in anucleate human erythrocytes in culture [15], suggesting that a PRDX-based clock, or an unidentified post-translational mechanism driving PRDX cycles, has the potential to define circadian time independently of transcription [16].

### 2.2. Comparative Circadian Clocks: Transcriptional/Post-Translational Oscillators

Notwithstanding the power of Kai-based and PRDX-based oscillations in prokaryotes, the prevailing view is that eukaryotic clocks incorporate more elaborate mechanisms, with a central involvement of transcription [2]. Forward mutagenesis paved the way for unravelling eukaryotic clockworks by screening for mutants with atypical circadian periods. This led to the identification of “core clock genes”, including *Period* (*Per*) in *Drosophila*, *Frequency* (*Frq*) in *Neurospora*, *Timing of cab expression* 1 (TOC1) in *Arabidopsis* and *Clock* in mouse. From these entry points, a series of biochemical and genetic approaches made it possible to define the core transcriptional components of the respective oscillatory mechanisms. The recurrent motif is one in which positive transcription factors drive the expression of genes encoding negative factors that, following a suitable delay, inhibit the initial activation. This closure of the negative feedback loop completes the first half of the circadian cycle, whilst the second half involves the progressive degradation of the negative factors to facilitate the re-initiation of a new transcriptional phase. Thus, the molecular basis to the oscillation can be viewed as a transcriptional/post-translational feedback loop (TTFL). In *Drosophila* the positive factors are Cycle and Clock, and the negative regulator Per is partnered by Timeless (Tim) [17], whereas the TTFL of *Neurospora* pivots around positive factors White-collar (WC)-1 and WC-2, which together drive the negative factor Frq [18]. The genetic network of the core clock in higher plants is more complex, incorporating the negative regulator TOC1 alongside at least eight other negative and positive transcription factors [19]. Similarly, in fruit flies and *Neurospora* additional accessory feedback loops stabilise and amplify the core oscillation within a broader transcriptional network. As with the post-translational prokaryotic clock of *S. elongatus*, these TTFLs engage with the general transcriptional apparatus to drive circadian waves of gene expression that direct daily cycles of cellular function and organismal physiology and behaviour. Consequently, cellular transcriptomes are highly dynamic and adaptive across circadian time.

### 2.3. The Intra-Cellular Circadian Clock Mechanism of Mammals

**Figure 1:**
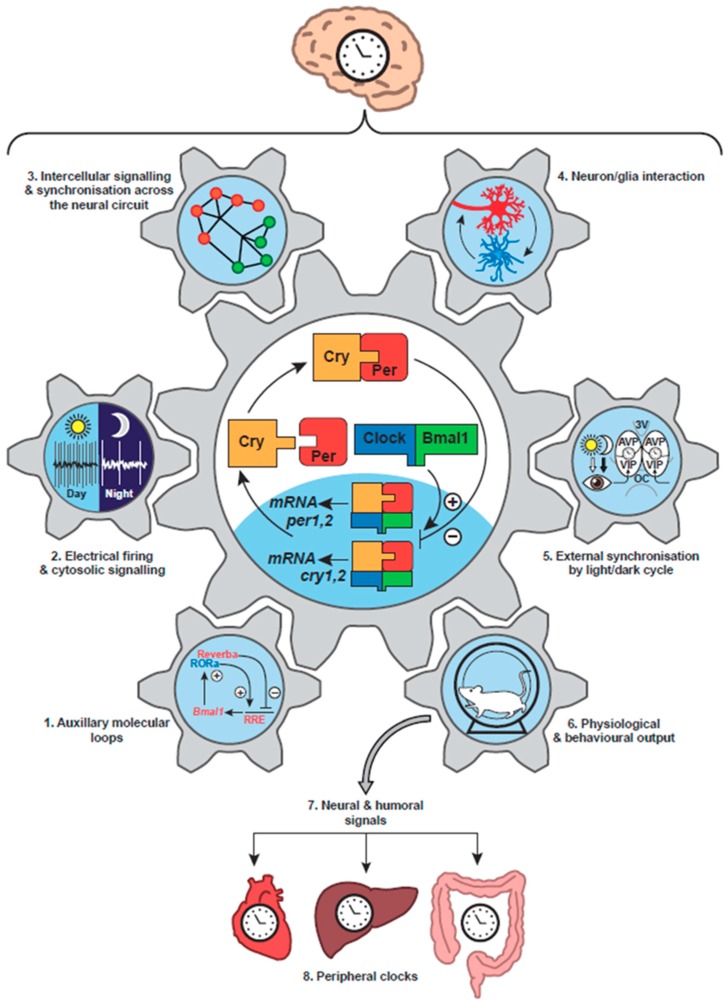
Schematic view of the mechanisms that direct circadian time-keeping in the suprachiasmatic nucleus. The suprachiasmatic nucleus (SCN) is the principal circadian pacemaker of the mammalian brain (upper panel) that ultimately orchestrates circadian metabolism and physiology by redirecting the activity of local, tissue-based circadian clocks (lowest panel). A characteristic feature of the SCN (central panel) is that outputs of the circadian timing system also operate as inputs to it. This recurrent activity, observed at all levels of organisation: molecular, cellular and whole-animal, confers on the SCN its markedly precise, high-amplitude and robust time-keeping. At the heart of the SCN time-keeper (central “cog wheel”) is the cell-autonomous core transcriptional/post-translational feedback loop (TTFL), in which with the negative factors Per associate with Cry to inhibit their own Clock/Bmal1-driven transcription. (**1**) This core oscillation is further stabilised by auxiliary intra-cellular molecular feedback loops, whereby TTFL-driven circadian expression of Rev-erb and Rora feeds back to control the transcription of Bmal1. (**2**) By its control of downstream clock-controlled output genes, the TTFL of the SCN drives circadian cycles of cell-autonomous electrical firing and associated cytosolic signalling, including daily rhythms of cAMP and [Ca^2+^]i, These in turn feedback into the TTFL via the calcium/cAMP-responsive elements of Per genes, adding further stability to the cell-autonomous SCN oscillator. (**3**) The cell-autonomous circadian rhythms are synchronised across the SCN circuit by inter-cellular signalling. Although the network topology of the SCN is undefined (represented here in cartoon fashion), neuropeptidergic cues are known to be critical: in their absence the cell-autonomous clocks desynchronise and lose amplitude. (**4**) SCN astrocytes also have circadian TTFL and cytosolic rhythms that are synchronised by, but differentially phased to, the neuronal TTFLs. Cues from astrocytes in turn can drive the cellular neuronal clocks, providing an additional level of reciprocal input/output signalling, further stabilising the system. (**5**) The ensemble oscillation of the SCN circuit is synchronised to solar time by retinal innervation, which activates VIP-expressing neurons of the core region of the SCN, and these in turn regulate the phase of AVP-expressing neurons of the SCN shell. (**6**) The circadian rhythm of neuronal activity in the SCN ultimately directs circadian patterns of behaviour and physiology via a network of yet-to-be-defined neural pathways emanating from the hypothalamus and brain stem. This TTFL-dependent output at the level of the whole animal can also act as a feedback onto the SCN TTFL, with increases in arousal acutely suppressing Per expression in the SCN, for example. (**7**) Circadian changes in behavioural and neuroendocrine state provide cues for internal synchronisation across the body. (**8**) These cues synchronise the cell-autonomous TTFLs within tissues to ensure that local circadian programmes of metabolism and physiology anticipate, and thereby adapt the organism to, the demands and opportunities of day and night.

The mammalian circadian clockwork has strong parallels with that of *Drosophila*. The positive factors are Clock and Bmal1 (a homologue of Cycle), which are basic helix-loop-helix transcription factors that heterodimerise via so-called PAS domains to bind DNA at Enhancer boxes (E-boxes) and thereby drive transcription [20] (Figure 1, centre). The negative factors Per1 and Per2 are homologues of dPer, the latter being the closer, but rather than pairing with Tim, they associate with Cryptochromes (Cry1, Cry2) to inhibit their own Clock/Bmal1-dependent transcription. In some invertebrates and higher plants, Cry proteins can be light-sensitive, reflecting their evolutionary origin from light–dependent DNA repair photolyases [21], but in mammals they are transcriptional regulators rather than photoreceptors. As with the TTFL of insects, fungi and plants, the core oscillation of mammals is embedded within a wider network. In this case, the Clock/Bmal1-dependent expression of nuclear receptors Rev-Erb and Rora feeds back to control the transcription of Bmal1 [22], conferring stability and precision to the timing network [23] (Figure 1, part 1). Furthermore, the final common pathway leading out from the oscillatory core involves a hierarchy of transcription factors, including the leucine zipper factors DBP and E4BP4 [24], that sustains circadian cycles of gene expression appropriate to the distinct daily metabolic and cellular programmes of specific tissues. Thus, early DNA microarray-based studies [25] and more recent RNA-seq analyses [26] have identified extensive circadian transcriptomes across mammalian tissues [27], which are translated into equally distinct circadian proteomes [28,29,30]. In this way, the metabolic capacity of cells changes predictably across circadian time and so generates and sustains adaptive daily rhythms. Importantly, the rhythmic metabolic outputs of the cellular circadian system may also feedback into the core oscillator. For example, hypoxia inducible factor 1 (HIF1), PRDX-dependent redox changes, NAD+-dependent sirtuins and haem (a co-factor to nuclear receptors) all influence the TTFL, supporting the view that the entire cell comprises a higher-level circadian time-keeper, with intermeshed and mutually supportive TTFL and metabolic/post-translational oscillations [31,32].

## 3. Circadian Clock Networks in Mammals

### 3.1. Tissue Localisation of Clocks

The paired suprachiasmatic nuclei (SCN) of the hypothalamus, sitting above the optic chiasm and flanking the third ventricle, are the central circadian pacemaker controlling behavioural rhythms in mammals [33]. In vivo and ex vivo, they exhibit a pronounced circadian cycle of spontaneous electrical firing, peaking in circadian daytime (Figure 1, part 2). Their ablation in experimental animals or their compression by vascular disease or pituitary tumours in human patients disrupts daily rest/activity patterns [34,35], and in lesioned animals intra-hypothalamic grafts of neonatal SCN tissue can rescue activity rhythms. Importantly, the period of restored behavioural rhythms is determined by the circadian genotype of the grafted tissue, not the host animal: definitive proof that the SCN is the behaviourally relevant clock [36,37]. Because of the focus on behavioural activity/rest rhythms and the difficulties in performing alternative cellular assays, the previously accepted view was that the retina (and perhaps the perinatal pineal gland) was the only extra-SCN tissue to house an intrinsic circadian clock in mammals [38]. This was reconsidered after the discovery, facilitated by the identification of mammalian clock genes, that most tissues and indeed fibroblasts in cell culture are able to express circadian cycles of clock gene expression. This was first demonstrated by biochemical analyses of serial time-course samples [39], but it was revealed in all of its elegance by real-time recording of bioluminescent and fluorescent reporters of clock gene expression in individual living cell [40,41]. The Per2::luciferase reporter mouse [42] and Per1-luciferase reporter rat [43] were particularly influential in this regard because they could be used to demonstrate independent circadian cycles of Per expression in various tissues isolated ex vivo in culture (Figure 1, part 8), and also probe the SCN-dependent mechanisms responsible for their entrainment in vivo. Furthermore, these approaches have been translated to primary human cells by using lentiviral vectors to introduce circadian reporter constructs and thereby demonstrate that human cells also contain cell-autonomous TTFLs with a molecular architecture comparable to that of rodents [44].

### 3.2. Internal Synchronisation of the Mammalian Circadian Timing System

If the identification of the core TTFL in mammals was the enabling breakthrough in understanding how an internal proxy of solar time is established, the discovery that it is present in all major tissues and active in innumerable cells revolutionised mammalian circadian biology [45]. It showed that the circadian clock system of mammals is very complex, with multiple levels of regulation, but that it is also hierarchical, with the SCN acting as the orchestrator of the system [33]. The TTFL of an SCN isolated as an organotypic slice culture can continue to oscillate, as revealed by bioluminescent reporters, with high precision and amplitude, effectively, indefinitely. In contrast, ensemble bioluminescence rhythms of other tissues rapidly damp over several days, as the cell-autonomous TTFLs lose amplitude and/or lose phase coherence across the tissue [41,42]. Ordinarily, SCN-dependent cues maintain phase order and amplitude of the peripheral tissue-based clocks [46] and so prevent damping. These cues may be endocrine, for example the SCN-dependent rhythm of corticosteroid secretion by the adrenal gland [3], or they may involve circadian changes in autonomic nervous function [24] (Figure 1, part 7). A particularly powerful synchronising cue is the daily cycle of feeding and fasting, which is an inevitable consequence of the cycle of sleep and wakefulness, as feeding does not occur during sleep. In an experimental context, shifting the time of feeding can produce a parallel shift in the phase of the TTFL of the liver and other tissues [47], and with it a phase shift of the hepatic circadian transcriptome and dependent metabolic functions [48]. In the short term, this may be an adaptive change to the daily physiological programme. In the setting of rotational shift-work, however, where eating schedules are continuously misaligned with light and darkness, sleep and wakefulness, destabilisation of internal circadian order is likely the cause of the increased susceptibility of workers to cardiovascular, metabolic and other systemic diseases, including cancer, revealed in epidemiological studies [49,50,51,52].

### 3.3. External Synchronisation of the Mammalian Circadian Timing System

Light is the principal stimulus for external synchronisation of circadian clocks, and in the context of mammals this is mediated via the direct retinal innervation of the SCN derived from the retinohypothalamic tract (RHT) [53] (Figure 1, part 5). The RHT is a monosynaptic pathway from a sub-category of retinal ganglion cells (RGC) that terminate on SCN retinorecipient neurons. The RHT also projects to a number of other sub-cortical, non-perceptual visual centres [54]. In these centres, as with the SCN, stimulus properties such as the overall intensity and the duration of luminance integrated over long time-scales (minutes) are more relevant than wavelength and spatial and temporal fine structure [55]. Notably, circadian entrainment persists in mice genetically deficient for the conventional cone and rod photoreceptors: a paradoxical finding at the time it was made [56]. Two significant discoveries resolved this paradox, however. First, using imaging of intracellular calcium, it was shown that in retinal explants lacking rod and cone function, a small population of retinal cells could be activated by light [57]. Second, homology-cloning studies of genes encoding G-protein coupled receptors (GPCR) from frog skin identified a novel vertebrate opsin: melanopsin. Moreover, the mammalian form of melanopsin was detected in primate and rodent RGCs [58], to which it confers cell-autonomous photoreceptivity [59]. Melanopsin is an invertebrate-like blue-light photoreceptor, causing a sustained and intensity-dependent depolarisation of cells in response to light [60]. It is therefore ideally suited to provide the stimulus properties required by the SCN for entrainment to the light/dark cycle. In the absence of melanopsin, rod and cone photoreception can maintain entrainment, but in the absence of the melanopsin-positive, intrinsically photoreceptive RGCs, entrainment is abolished [61]: the RHT is the necessary final common pathway to the SCN and its dependent circadian network of peripheral clocks.

## 4. Neurobiology of the Suprachiasmatic Nucleus

### 4.1. Cellular Organisation of the SCN

**Figure 2:**
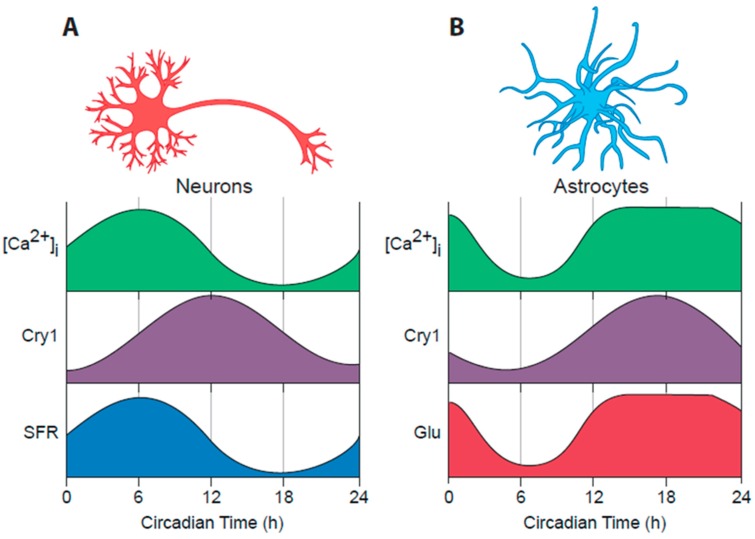
Schematic view of the circadian programmes of SCN neurons and astrocytes. (**A**) SCN neurons exhibit pronounced circadian rhythms of [Ca^2+^]i, as measured by a genetically encoded fluorescent reporter. These can be phased-mapped to the TTFL by simultaneous bioluminescent recording, in this case of Cry1-luciferase, and to the circadian rhythm of spontaneous firing rate (SFR). SFR and [Ca^2+^]i, peak simultaneously in mid circadian day, in advance of Cry1 (and Per, not shown) transcription. (**B**) SCN astrocytes also exhibit circadian cycles of [Ca^2+^]i, and TTFL activity (Cry1-luciferase) which differ from those of neurons on the basis of their contrasting phases (delayed in astrocytes) and waveforms (broader calcium peak in astrocytes). The circadian cycle of extracellular glutamate (Glu) is phase-locked to the calcium rhythm of astrocytes and shares its waveform. The quasi-anti-phasic patterning of neuronal and astrocytic rhythms likely reflects their mutually supportive interplay, which by appropriately timing release of inter-cellular mediators coordinates their respective cellular activities and thereby adds stability to timekeeping in the SCN.

Studies with dispersed SCN cultures suggest that most, if not all, SCN neurons can express cell-autonomous circadian rhythms of action potential firing and intracellular calcium ([Ca^2+^]i), which in turn are dependent on the cellular TTFL [62] (Figure 2A). Moreover, all SCN neurons express the inhibitory neurotransmitter GABA [53]. There is, nevertheless, considerable neurochemical diversity across the 10,000 or so neurons of a single SCN, reflected principally in their neuropeptidergic co-transmitters. Conventionally the SCN is divided into “core” and “shell” sub-domains, the core containing cells that express vasoactive intestinal peptide (VIP) or gastrin-releasing peptide (GRP) whereas the shell contains cells that express arginine vasopressin (AVP) (Figure 1, part 5). A number of other neuropeptides; neurotensin, somatostatin, prokineticin2 (Prok2) are expressed in cells across the SCN, respecting or straddling these sub-divisions [53,63], but the principal distinguishing feature is that the core SCN is the site of RHT innervation. Entrainment by light-dependent activation of the RHT terminals is mediated by glutamatergic and peptidergic (PACAP) stimulation of VIP and GRP cells in the SCN core [64]. This is followed by a second intra-SCN stage involving GABA-, VIP- and GRPergic signalling from core to shell neurons [33]. Correspondingly, the loss of receptors for VIP or GRP attenuates SCN and behavioural responses to light, whilst pharmacological blockade of GABA signalling loosens core-to-shell coupling, as does loss of receptors for AVP [65,66]. Neurons in both the core and shell send efferent projections from the SCN to distal targets, which are predominantly in the hypothalamus, midline thalamus and brain stem [33]. Some projections, such as those to orexinergic/ hypocretin neurons of the lateral hypothalamus, provide an obvious route for the circadian control of sleep/wake and/or feeding cycles, whilst innervations of the brain stem and the paraventricular nucleus of the hypothalamus offer routes for circadian control of the pituitary/adrenal axis, and pineal melatonin secretion, respectively [67] (Figure 1, part 6). GABA, VIP and Prok2 [68,69] are important mediators of these circadian outputs, but overall the way in which specific circadian cues are conveyed from the SCN to most downstream targets to control physiology and behaviour is poorly understood, not least because most pathways are multi-synaptic and indirect.

### 4.2. Cellular Oscillations in the SCN: Electrical, Transcriptional and Metabolic

The advent of real-time imaging approaches, using bioluminescent and fluorescent reporters of cellular functions, has provided an entirely new view on the circadian programme of the SCN, both ex vivo as organotypic slice cultures and more recently in vivo in freely moving mice [70,71]. Per expression in the SCN is high in circadian day, when spontaneous firing of action potentials is also high, and low in circadian night, when most SCN cells are hyperpolarised and electrically quiescent [62] (Figure 1, part 2). These daily cycles are accompanied by equally pronounced rhythms of neuronal intracellular calcium [Ca^2+^]i [72], neuronal membrane potential [73] and intracellular cAMP levels [74], all of which peak simultaneously, approximately 4–6 h in advance of Per and Cry expression (Figure 2A). The genes encoding Per1 and Per2 carry cAMP/calcium-response regulatory elements (CREs), and so it is likely that the rhythm of spontaneous electrical firing is coupled to the Per-dependent TTFL via the changes in [Ca^2+^]i and cAMP: indeed CRE-driven transcription, reported by a CRE-luciferase lentivirus vector, peaks in SCN slices shortly in advance of Per1 [72]. In turn, the TTFL will direct rhythms in the expression of ion channels and other factors that sustain rhythms of electrical activity [75,76,77,78]. This reciprocal coupling of electrical firing and transcription likely contributes to the stability of the SCN clock mechanism, and the metabolic demands of such rhythms are reflected by a circadian cycle of PRDX superoxidation, which peaks in the SCN in circadian night [14]. The extent to which PRDX and other cytosolic oscillations in turn regulate the SCN TTFL remains to be determined [79], but if so, this feedback may represent a further source of stability for the SCN cellular clockwork [80].

### 4.3. Entrainment of the SCN TTFL Cellular Clocks

Resetting of the cellular TTFLs is dependent on acute induction of electrical activity and accompanying expression of Per1 and Per2 [75]. When Per induction occurs in early circadian night (dusk) as Per expression is naturally declining, the acute induction delays the ongoing TTFL oscillation, whereas acute induction in late circadian night (dawn), when Per expression is starting to increase, accelerates the increase and thereby advances the TTFL clock [81]. Light presented in circadian day, when firing rates and Per expression are already maximal, has little effect on the TTFL: the clock gates the sensitivity of the TTFL to retinal input. These phase-dependent effects of light on the SCN TTFLs, with small daily advances or delays following nocturnal light pulses, sets the period of the SCN clock to exactly 24 h and also synchronises the phase of the TTFL to solar time. In turn, circadian behavioural and endocrine rhythms are synchronised to the light/dark cycle by small daily shifts caused by dusk or dawn light. The necessary induction of Per expression in core neurons is likely via CREs [82], activated by increases in [Ca^2+^]i triggered by glutamatergic NMDA and AMPA receptors in the core and by [Ca^2+^]i and cAMP signalling by peptidergic GPCRs on shell neurons [72]. In addition, resetting light pulses acting via AMPA and NMDA signalling induce the immediate-early transcription factors Fos and phospho-Jun in core neurons [83]. These transcription factors may then hetero-dimerise and sustain Per expression via their AP-1 target sequences in Per (R.G. Foster, pers. comm.). In the shell, the second stage of resetting involves signalling by VIP via the VPAC2 GPCR to induce a network of transcriptional responses associated with Per induction [84]. Signalling via ERK1/2 and tuning by its negative regulator DUSP4 are critical elements of this VIP-directed circadian re-setting. Loss of the VPAC2 receptor compromises the circadian regulation of sensitivity to RHT input: light is able at all phases to induce Per and Fos expression, and activate Ca^2+^-dependent kinases [85]. Conversely, the power of VIP-mediated signalling is highlighted by the ability of a VIP-competent SCN slice to initiate and sustain, in a co-culture configuration, the circadian TTFL of an initially arrhythmic VIP-deficient SCN [86]. Whereas glutamatergic and GABAergic signalling in the SCN is rapid and synaptic consistent with acute resetting, it is likely that VIP (and other) peptidergic signalling is slow and paracrine in nature: properties consistent with the broad spatio-temporal domain of the SCN TTFL clockwork. 

## 5. The Suprachiasmatic Nucleus as a Neuronal Network

### 5.1. Synchronisation across the SCN Circuit

Imaging at the level of single cells and high throughput electrophysiological approaches have made it possible to explore the circadian behaviour of individual SCN cells in the context of the wider circuit. A powerful emergent property of the SCN is the synchrony of the individual cells, which is critically important if the circuit is to broadcast to the animal an explicit, unified time signal. It should be noted, however, that cellular activity across the SCN (be that TTFL or electrical) is not simultaneous. Rather, sub-regions of the SCN are differentially phased, with the effect that in imaging studies waves of cellular activity can be seen to “wash” across the SCN circuit in a stereotypical trajectory, dorsomedial to ventrolateral [70,72]. The mechanisms that establish this progressive phase order and its relevance to circadian outputs are not clear, although it may contribute to the encoding of daylength, insofar as the phase dispersal is greater in SCN from animals exposed to longer (summer-like) photoperiods. There is some evidence that antagonistic VIP- and GABAergic signalling is important in sculpting the photoperiodic wave, but in the absence of photic entrainment the phase dispersion contracts back to the default observed in animals under a 12:12 light:dark cycle [87].

The steady-state synchrony across the circuit is critically dependent on VIP signalling, insofar as loss of VIP or the gene encoding VPAC2, the cognate receptor for VIP in the SCN, leads to the complete loss of cellular synchrony in the SCN slices, and concomitant disruption of circadian behavioural and endocrine rhythms in the animal [85,88]. Furthermore, acute optogenetic activation of VIP neurons can entrain circadian TTFL and behavioural rhythms [89] whilst sustained chemogenetic activation of VIP neurons can re-programme the spatio-temporal wave [72]. As noted above, co-culture studies have demonstrated a powerful paracrine action of VIP in circuit-level synchronisation. Within the shell, AVP signalling is important for coupling across the circuit, such that loss of the two GPCRs for AVP expressed in the SCN does not compromise circadian time-keeping, but it does render the more loosely coupled SCN circuit more labile to resetting stimuli [66]. Consequently, behavioural activity rhythms adjust to altered lighting schedules far more rapidly. Consistent with a paracrine role for AVP, in co-culture studies where the VPAC2 GPCR is absent and so any effect of VIP-dependent signals is excluded, an SCN graft can nevertheless drive the TTFL of a VPAC2-null SCN, and antagonists to the AVP GPCRs block this paracrine effect [86]. A future goal, therefore, is to understand the cellular, neurochemical and anatomical mechanisms that confer on the SCN circuit the critical emergent properties of synchrony and phase dispersion, and to relate those properties to the orchestration of the body-wide circadian network. To achieve this requires knowledge of the topology of the SCN network (Figure 1, part 3).

### 5.2. Are There Pacemaker Cells in the SCN?

A second emergent property of the SCN circuit is its ensemble period. At a cell-autonomous level, circadian period is a property of the TTFL generated, in ways we do not yet understand, by the interacting time constants of the various molecular (transcription, translation, degradation) and cellular (nuclear localisation of complexes, metabolic inputs) stages. For example, mutations of Per and Cry, and their respective degradation pathways (kinases, ubiquitin ligases) that enhance or decrease protein stability lead, respectively, to longer and shorter periods for the TTFL and corresponding behavioural and endocrine rhythms, in both experimental animals and humans [33]. The astonishing robustness of the cellular and circuit mechanisms is illustrated by the maintenance of cellular and circuit-level integrity in the face of extreme genetic and pharmacological perturbations: even when driven to oscillate with periods of below 17 h or above 42 h, the circuit remains as well organised as when it oscillates at 24 h [90]. Under physical or pharmacological isolation, however, individual SCN cells express a range of free-running periods that deviate around the ensemble mean. Within the circuit, therefore, there must be some form of computation, an interplay, that draws all cells to a common period and this raises the question of whether some cells contribute more than others to that computation: are there “pacemaker” cells within the SCN. Do particular cells or cell groups determine the ensemble period, reflective of their own cell-autonomous period, or is the computation of ensemble period a devolved “democratic” process?

A series of studies have addressed this by testing the effect of altering the cell-autonomous period of distinct SCN neuronal populations. Genetic lengthening of the period of SCN AVP cells leads to a corresponding lengthening of behavioural activity rhythms in mice, indicative of a pacemaking activity, but when the SCN of such mice is isolated in slice culture the effects on period are rapidly dissipated [91,92]. A more comprehensive pacemaking effect is evident when neuromedin S (NMS)-positive cells are targeted: at the levels of the SCN TTFL and overt behaviour, ensemble circadian period is determined by the cell-autonomous period of these neurons [93]. Furthermore, if synaptic signalling by NMS cells is blocked by conditional expression of tetanus toxin, control of circadian behaviour is lost. A strong case can therefore be made for NMS neurons as behaviourally relevant pacemakers. The specificity of this role, however, remains unclear because NMS cells comprise the majority of SCN neurons, including VIP and AVP cells, and NMS itself is not necessary for normal circadian function. The pacemaking by NMS cells may therefore arise from a quantitatively, rather than a qualitatively, distinct contribution. A further intriguing insight into ensemble computation is provided by studies in mice in which the cell-autonomous period of cells expressing the dopamine 1a receptor (Drd1a) is lengthened by 4 h relative to non-Drd1a cells [94]. Drd1a cells include most SCN VIP cells and ca. 50% of AVP cells, and overall about 50–60% of the SCN neurons. Consistent with this, the periods of the SCN TTFL and of behavioural activity rhythms are correspondingly lengthened when Drd1a cells are targeted. However this effect is not complete, because in a minority of cases (ca. 30% of SCN and mice) periods are not lengthened, suggestive of a pacemaking role for non-Drd1a cells. More intriguing still, in ca. 10% of mice and SCN the circadian period alternates on an apparently stochastic basis between stable long and short periods, possibly reflecting on ongoing circuit-level computational struggle between Drd1a and non-Drd1a cells to set ensemble period. The nature of the computation is to be determined: how do cells interact to determine and then adopt a common circadian period? It is clearly a powerful property of the circuit because it is able to establish a single ensemble period even though the composite cells have periods that contrast by 4 h. Again, whether Drd1a cells have a quantitative or a qualitative pacemaking role is unclear, but their central role in the clock is reinforced by the observation that their optogenetic activation is sufficient to entrain the SCN TTFL and behavioural rhythms [95].

### 5.3. Exploring SCN Cell-Autonomous and Network-Level Timing by Genetic Complementation

Genetic complementation, which has been widely applied in cell culture models where transfection of DNA constructs is routine [96], provides an alternative way to explore the determinants of cell-autonomous and network-level timing in the SCN. The approach uses a clock-deficient mouse or tissue, typically a Cry1/Cry2 null mutant, and then introduces Cry protein and tests, with molecular definition, what aspects of circadian time-keeping can be initiated. The genetic access afforded by AAV-based viral vectors has made it possible to interrogate the properties of Cry proteins within the SCN TTFL by expressing them in a Cry-null background, but with one caveat. Cry-null adult mice and SCN slices are completely arrhythmic because the Cry-dependent TTFL is deficient. In contrast, in some neonatal SCN (ca. 30%), transient circadian oscillations can be observed, albeit with atypical short (ca. 18 h) periods and rapid damping [86,97]. The presence of these cycles is intriguing: do they represent a TTFL driven solely by Per-mediated negative regulation, and/or, does a cryptic cytosolic oscillator reminiscent of the PRDX cycle of erythrocytes [98], or rhythmic ion-fluxes [99] drive the cycles of Per-reported bioluminescence?

Regardless of their origin, these rhythms, when present, dissipate rapidly and so arrhythmia of SCN slices can be confirmed before initiating AAV-mediated complementation. Such complementation can then provide important insights into SCN function. The first is that in SCN slices that have never encountered Cry proteins, the virally mediated expression of Cry1 or Cry2 can immediately (within 36 h) initiate stable, high amplitude circadian TTFL oscillations [100]. Cry proteins are therefore essential clock components but they are not required to specify the development of circadian competence. Second, the individual protein determines the period of the oscillation: when Cry1 or Cry2 are expressed in the same phase from the same minimal Cry1 promoter, they establish slow (>26 h) or fast (ca. 22 h) oscillations, respectively, confirming their distinct roles in the TTFL [101]. Further, the correct phase of expression of Cry is critical if it is to engage with and drive the TTFL: Cry1 expressed in circadian night rather than circadian day under by the Bmal1 promoter is unable to initiate a stable TTFL. Equally, when Cry1 is expressed constitutively under the synapsin promoter it is equally ineffective. Thus, driving the TTFL relies on the intrinsic properties of Cry proteins and their phase of expression. Beyond these cell-autonomous effects, complementation with Cry protein reveals circuit-level determinants of the SCN clock. Expression of AVP is low in Cry-null SCN, and this increases on AAV-mediated Cry1 expression. This increase in AVP expression is an important factor in initiating circuit-level rhythms because pharmacological blockade of the two GPCRs that mediate AVP signalling in the SCN prevents the initiation of the TTFL [100]. Blockade once the oscillation is established is, however, without effect. This highlights a central role for Cry-dependent expression of AVP as a mediator of the cell-autonomous effects of Cry1 on circuit-level control.

### 5.4. Translational Switching as a Novel Means to Explore SCN Circadian Timing

**Figure 3:**
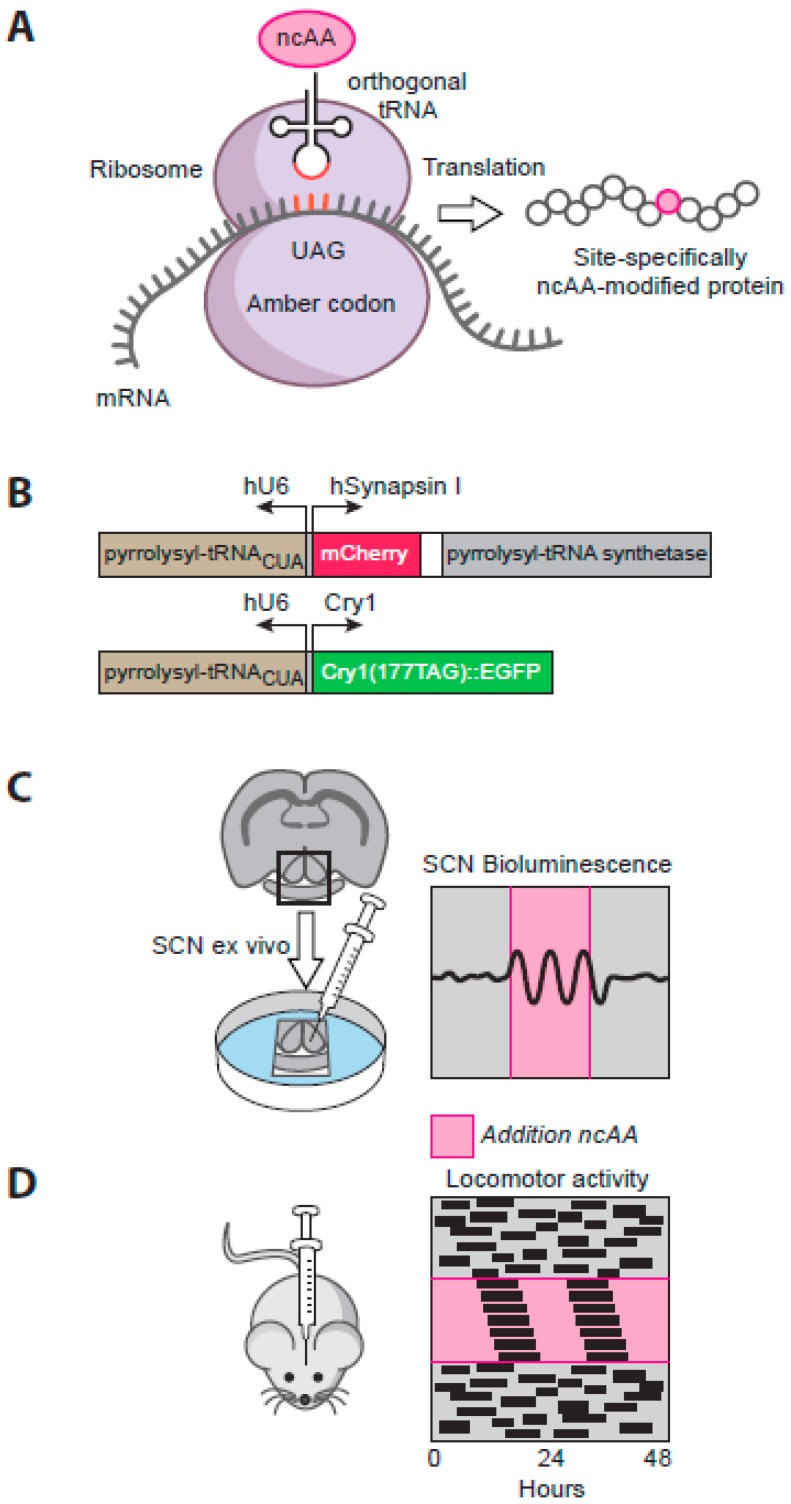
Translational switching: a versatile tool for reconstituting and exploring the circadian clock. (**A**) Schematic view of the site-specific incorporation of a non-canonical amino acid (ncAA, pink shade) during translation of a protein by using a novel aminoacyl transfer RNA (tRNA) (charged by a novel tRNA synthetase) that recognises the amber stop codon, thereby allowing translational read-through. **(B**) Constructs encoding the orthogonal tRNA and tRNA synthetase, alongside the amber-sensitive Cry1::EGFP, both delivered to SCN cells by AAV vectors. (**C**) In arrhythmic Cry-null SCN slices, pre-transfected with the two AAV vectors, delivery of ncAA (pink shade) initiates a circadian rhythm (reported as Per2-driven bioluminescence), which is lost immediately on withdrawal of ncAA. (**D**) In arrhythmic Cry-null mice, pre-transfected with the two AAV vectors directed at the SCN, delivery of ncAA (pink shade) in drinking water initiates a circadian behavioural rhythm, which is lost immediately on withdrawal of ncAA.

Remarkably, the effects of complementation observed in the SCN slice ex vivo are replicated by targeting the SCN of Cry-null mice in vivo [102]. Within a few days after stereotaxic injection of AAV encoding Cry1 under the minimal Cry1 promoter, previously arrhythmic mice exhibit robust circadian behavioural rhythms, with a definitive long period indicative of their control by the recombinant Cry protein. Thus, in animals where the SCN contains the only molecularly competent circadian TTFL, organised circadian behaviour can nevertheless be imposed. As with the local SCN circuitry, this shows that all of the neural pathways across the brain and spinal cord necessary for the SCN to control behaviour are specified independently of Cry protein expression. Making the expression of Cry protein conditional has further extended the potential of this approach. By re-engineering prokaryotic tRNA and tRNA synthetase pairs, it is now possible to develop genetic code expansion as a means to incorporate orthogonal, non-natural amino acids (ncAA) at an ectopic amber stop codon in proteins of interest [103] (Figure 3A). In this way, expression of Cry1 can be made conditional on supply of the ncAA alkyne lysine (AlkK) to SCN culture medium or mouse drinking water [102]. Further conditionality is provided by cell type-specific expression of the necessary tRNA/ tRNA synthetase pair, delivered by AAV vectors (Figure 3B). In arrhythmic Cry-null SCN slices or mice expressing the tRNA synthetase solely in neurons under the synapsin promoter, provision and removal of AlkK can reproducibly and reversibly initiate Cry1 expression, and thereby initiate TTFL and behavioural circadian rhythms, with long periods definitive of a Cry1-driven clock [102] (Figure 3C, D). Thus, SCN neurons alone are sufficient to control circadian behaviour in an otherwise clockless mouse. In addition, by titrating the concentration of AlkK, and thus the level of expression of Cry1, it has been possible to show that the period of the SCN TTFL bioluminescence rhythm is determined, dose-dependently, by the level of neuronal expression of Cry1. Translational switching thereby offers considerable scope for future quantitative analysis of the molecular circadian mechanism, whilst beyond that the rapid (within 48 h) initiation and reversibility of molecular and behavioural circadian rhythms shows the circadian network to be highly stable in its steady-state operation, but very responsive to changes in its molecular components. Given that the circadian behavioural cycle will drive a corresponding circadian cycle of feeding in an otherwise clockless mouse, this preparation also provides a useful platform for future interrogation of the dynamic mechanisms mediating feeding/fasting-dependent co-ordination of the periphery by the SCN.

## 6. Astrocytes as Clock Cells: The “Dark” Side of the SCN Timing Network

### 6.1. Circadian Properties of SCN Astrocytes

This discussion has focused on the role of SCN neurons in the circadian system of mammals, but the SCN also contains abundant astrocytes, glial cells considered to be important in controlling cerebral vasculature and the delineation of synapses [104]. Indeed, expression of the astrocytic cytoskeletal factor glial fibrillary acidic protein (GFAP) exhibits a circadian variation in the SCN that may be related to movements of astrocytes surrounding synapses containing RHT terminals [105]. With the development of genetic access to astrocytes via AAV-expressed constructs, a more directed analysis of the circadian properties and functions of astrocytes is now possible. As noted above, the circadian rhythm of neuronal [Ca^2+^]i peaks in circadian daytime, around circadian time (CT)06. Simultaneous recordings of neuronal and astrocytic [Ca^2+^]i in the same SCN slices, using red and green fluorescent reporters driven by synapsin and GFAP promoters respectively, yielded startling results [73]. First, SCN astrocytes exhibit a strong circadian cycle of [Ca^2+^]i: astrocytes are circadian cells just as are neurons, but the peak of the [Ca^2+^]i rhythm in astrocytes was much broader than the sharp peak of neurons (Figure 2B). More importantly, however, it was phased to circadian night, ca. CT18, indicating that the activity cycle of astrocytes in the SCN runs in anti-phase to that of the SCN neurons. The presence of a TTFL in astrocytes had previously shown in both cortical and SCN astrocytes [106,107], but when a recombinase-dependent, Cry1-luciferase was expressed either in neurons or astrocytes and phase-mapped to internal neuronal [Ca^2+^]i, it was revealed that differential phasing between the circadian programmes of SCN neurons and astrocytes also existed at the TTFL level, with Cry1-luciferase in astrocytes peaking in circadian night (Figure 2B). Surprisingly, therefore, the SCN circadian network incorporates two functionally distinct cellular populations: day-active neurons and night-active astrocytes harnessing a differentially phased TTFL [108] (Figure 1, part 4).

### 6.2. SCN Astrocytes Control SCN Molecular Rhythms and Behavioural Rhythms

**Figure 4:**
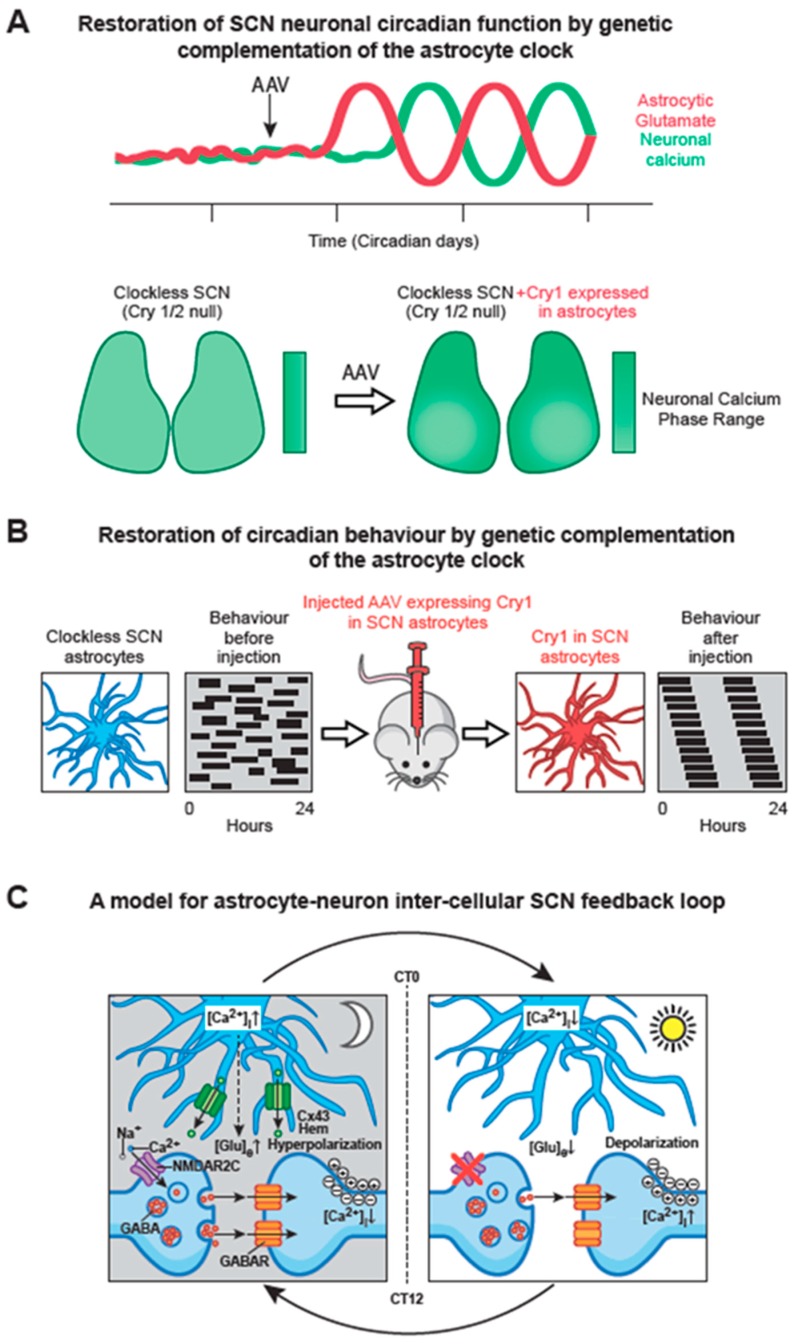
Astrocytic-neuronal interplay in the generation of SCN circadian rhythms: a synthetic model. (**A**) Schematic illustration that AAV-mediated, selective expression of Cry1 in astrocytes of arrhythmic Cry1/2-null SCN is sufficient to initiate circadian rhythms of neuronal [Ca^2+^]i levels (green) notwithstanding the absence of a neuronal TTFL in these SCN. In parallel the newly circadian-competent astrocytes also initiate a circadian rhythm of [Glu]e (red), phased appropriately to peak in circadian night. Real-time imaging of slices further reveals that the astrocytically initiated calcium rhythm in neurons expresses the appropriate emergent spatio-temporal wave across the SCN circuit. (**B**) Selective expression of Cry1 in astrocytes of the SCN, achieved by stereotaxic injection of AAV vectors in arrhythmic Cry1/2-null adult mice, is sufficient to initiate and support circadian oscillations of locomotor behaviour. This likely reflects initiation of the SCN neuronal rhythms via restored rhythmic glutamate signalling from astrocytes, and subsequent engagement of downstream motor centres by the driven neurons. The long period of the restored rhythm is reflective of the cell-autonomous rhythm of Cry1-activated SCN astrocytes. (**C**) A model of astrocytic–neuronal interaction. During circadian night (left), extracellular glutamate concentrations ([Glu]e) are high, driven by astrocytic release of glutamate via Cx43 hemichannels and glutamate transporters *(*not shown). This activates presynaptic NR2C subunit-containing NMDA-type glutamate receptors (NMDAR2C), increasing the presynaptic intracellular Ca^2+^ concentration ([Ca^2+^]i) to facilitate GABA release, and thereby suppressing network-wide electrical activity of postsynaptic neurons. During circadian daytime (right), clearance of extracellular glutamate by reduced astrocytic glutamate release relieves GABAergic tone across the network, leading to depolarization and increased electrical firing of SCN neurons. Redrawn from [73].

To test the importance of astrocytes in SCN time-keeping, the cell-autonomous period of astrocytes or neurons was lengthened by 4 h by local deletion of a floxed, short-period allele of casein kinase [109], using AAV-expressed Cre driven by either synapsin or GFAP promoters. Neuronally specific deletion lengthened behavioural period as anticipated, but more surprisingly so did astrocytically specific targeting. Thus, long-period (24 h) SCN astrocytes can impose their cell-autonomous period on circadian behaviour even when it conflicts with that of the (faster, 20 h) neuronal TTFL [73]. A similar conclusion came from a different experimental configuration, in which a genomically encoded, astrocytically specific recombinase was used to delete a single copy of the short-period allele [107] and again the period of behavioural activity rhythms was lengthened accordingly (by 2 h), an effect also evident in the SCN taken from the adult mice. To test whether a competent TTFL in astrocytes alone is sufficient to control SCN molecular rhythms and circadian behaviour, the complementation approach described above was applied, using AAVs encoding synapsin- or GFAP-driven recombinase, partnered with a recombinase-dependent version of the AAV-expressed Cry1. Neuronally specific expression of Cry1 in Cry-null SCN slices and in Cry-null mice, initiated molecular and behavioural rhythms as anticipated. More surprisingly, a comparable effect was seen in SCN slices and adult mice when Cry1 was expressed solely in SCN astrocytes [108] (Figure 4A, B). Thus, circadian competent SCN astrocytes are able to drive the otherwise circadian-incompetent SCN and thereby direct overt behavioural rhythms. Importantly, the astrocytically dependent rhythms were of a long period, confirming the definitive role of the locally expressed Cry1, but they were nevertheless shorter than the neuronally specified rhythms. In addition, in the SCN slices where the progressive initiation of TTFL cycles could be monitored, it was clear that neuronally expressed Cry1 initiated rhythms faster than did astrocytically expressed Cry1. The differential time-course likely reflects the fact that astrocytes ultimately exert their actions indirectly via changes in neuronal function. Nevertheless, these studies show that astrocytes are not merely passive supporting cells in the SCN: they are equal partners with neurons in the circadian timing network on the SCN, with distinct circadian properties.

### 6.3. Circadian Signalling from SCN Astrocytes to SCN Neurons

By what means might SCN astrocytes convey their intrinsically generated circadian time-cues to SCN neurons? It is now evident that astrocytes release a large number of neuroactive compounds and that these “gliotransmitters” perform regionally specific roles in circuits across the brain [104]. Glutamate is the principal transmitter of the neuronal RHT, but commonly it is also a gliotransmitter. In the SCN slice, RHT inputs are excluded and the neuronal circuit is predominantly GABAergic. Nevertheless real-time imaging with iGluSnFR, a fluorescent reporter of extracellular glutamate levels ([Glu]e), revealed not only that [Glu]e can be detected, but also that it exhibits a very pronounced circadian oscillation [73] (Figure 2B). Even more remarkably, the oscillation is in phase with that of astrocytic [Ca^2+^]i, peaking in circadian night when neuronal activity is at its nadir, and with an extended peak waveform comparable to that of astrocytic calcium. These data are consistent with the circadian release of glutamate arising from nocturnally active astrocytes, a view supported indirectly by the observation that in Cry-null SCN the [Glu]e rhythm is absent but it can be induced by astrocytically expressed Cry1, peaking in anti-phase to neuronal calcium [108] (Figure 4A). Astrocytes release some gliotransmitters via membrane- spanning hemichannels, formed from multimeric complexes of Connexin 43, which is highly expressed in SCN astrocytes. Consistent with a circadian role, pharmacological blockade of connexin hemichannels in wild-type SCN slices causes rapid and reversible damping of the TTFL, reported by Per2-luciferase bioluminescence. Furthermore, it also stops the circadian oscillation of [Glu]e in Cry-null SCN in which recombinant Cry1 is expressed solely in astrocytes. This loss of the [Glu]e rhythm is accompanied by damping of the TTFL bioluminescence rhythms in such slices [108].

A view is emerging, therefore, that nocturnally active astrocytes release glutamate via Cx43 hemichannels, and the circadian cycle of [Glu]e is important for driving circuit-level circadian oscillations (Figure 4C). Indeed, sustained elevation of [Glu]e by pharmacological blockade of glutamate transporters on neurons and astrocytes rapidly compromises the SCN TTFL [73], but by what mechanism might glutamate control SCN neurons? The RHT signals via NMDA receptors containing NR2A and NR2B sub-units expressed on core SCN neurons, but the dorsal shell SCN mainly expresses NMDA NR2C sub-units. Pharmacological blockade of these NR2C receptors desynchronises the individual cell-autonomous TTFLs and damps the ensemble TTFL of the SCN slice, distorting the spatio-temporal waves of clock gene expression. This unexpected finding highlights the role of NR2C–containing complexes in circuit synchronisation, independently of any effects of the RHT. A combination of electrophysiological recordings and imaging of pre- and post-synaptic [Ca^2+^]i supports a model whereby pre-synaptic NR2C-containing NMDA receptors, triggered by release of glutamate in circadian night, depolarise GABAergic terminals and thereby enhance inhibitory GABAergic tone across the SCN [73]. As astrocytic release of glutamate declines in circadian daytime, GABAergic tone is reduced, neurons depolarise and spontaneous firing increases, engaging with the cell-autonomous TTFL, causing a daytime peak of Per expression as described earlier. This model resolves a long-standing paradox in SCN function: how can firing rates spontaneously increase across an exclusively GABAergic circuit, in which higher firing would cause greater release of inhibitory GABA and so damp the circuit? By devolving circadian control of GABAergic tone away from neurons to astrocytes, the circuit can readily progress from states of daytime neuronal activity and night-time neuronal quiescence (Figure 4C).

## 7. Conclusion: Future Prospects

As noted above, the elucidation of the molecular genetic basis of circadian time-keeping is a landmark achievement in biology and was the product of many laboratories, leading to numerous personal and institutional rewards, and culminating in the award of the Nobel Prize for Physiology or Medicine in 2017 to Hall, Rosbash and Young who elucidated the Per-based negative feedback loop mechanism in *Drosophila* [110]. What next? Understanding the circadian mechanisms that so clearly underlie the control of growth, metabolism, disease-resistance, etc. in prokaryotes and higher plants provides innumerable opportunities for advances in biomass generation, bio-pharmaceutical synthesis and the enhancement of crop-yield. In the context of mammals, having shown how deeply wired the clock sits within all cellular and physiological processes, the field can now start to highlight which “circadian levers” can be pulled to mitigate, manage and possibly even reverse systemic disease. An early advantage has been to better engineer lighting environments and electronic displays to minimise disruption of the clock and sleep from inappropriate activation of the RHT [111]. More generally, by mapping circadian transcriptomes and proteomes, it has become evident that many diseases are affected by circadian mechanisms, and equally that many common medications have clock-dependent molecular targets [27]. From a neurobiological perspective, the SCN remains the focus of attention. We need to understand its network-level operations if we are to appreciate correctly its definitive function as the circadian pacemaker. We have no knowledge of the topology of the circuit—does it have small-world properties for example. What computations does it make, and to what degree does it consist of specialised nodes and super-connected hubs? Although neuropeptidergic identity continues to be a useful proxy for cell and circuit-level functions, it may well be that we need a deeper understanding of the natures of SCN cells, both neurons and astrocyte, which may well be orthogonal to the conventional view of core, shell, GABA and peptide. Recent developments in single-cell transcriptional analyses offer a ready route to this higher-order, topologically refined understanding [63] especially when combined with anatomically explicit synaptic mapping. Indeed, assembling the SCN connectome could well be an important enabling stage, a test-bed in growing efforts to understand more extensive mammalian connectomes.

Finally, in this whole-brain context, elucidation of “the clock” now makes it possible to embark on a more mechanistic analysis of the control of sleep and wakefulness. This has always been a larger prize, and indeed it could be argued that until neuroscience understands the nature, the function and the control of sleep, it will not have succeeded in its highest ambitions. Significant advances in understanding the molecular and genetic basis to sleep are coming from studies of *Drosophila* [112,113], and even though the neural circuitry directing sleep in flies may differ from that of mammals, the precedent of circadian timekeeping shows that molecular and genes may well be conserved. At a neural circuit level, by following the routes whereby the SCN clock controls the timing of sleep, it should be possible to map neural and neurochemical pathways pivotal to the initiation, maintenance and termination of sleep and wakefulness. Given that sleep and wakefulness are expressed coherently across the neuraxis, and it is clear that many brain areas host local circadian clock mechanisms, the model of a hierarchical clock network revealed by studies of the periphery may well be directly relevant to the control of sleep and wakefulness. In this context, misalignment of local cerebral clock mechanisms may therefore be a critical feature of psychiatric and/ or neurological diseases. Bringing the dimension of circadian time into medicine is an enormous challenge, but one that offers equally enormous and unprecedented opportunities for human advancement.
